# Interpersonal counselling versus perinatal-specific cognitive behavioural therapy for women with depression during pregnancy offered in routine psychological treatment services: a phase II randomised trial

**DOI:** 10.1186/s12888-021-03482-x

**Published:** 2021-10-15

**Authors:** Jonathan Evans, Jenny Ingram, Roslyn Law, Hazel Taylor, Debbie Johnson, Joel Glynn, Becky Hopley, David Kessler, Jeff Round, Jenny Ford, Iryna Culpin, Heather O’Mahen

**Affiliations:** 1 Population Health Sciences, University of Bristol, Bristol, UK; 2 Anna Freud Centre, London, UK; 3 University Hospitals Bristol and Weston NHS Trust, Bristol, UK; 4 Psychology, College of Life and Environmental Sciences, University of Exeter, Exeter, UK

**Keywords:** Depression, Pregnancy, Antenatal depression, Prenatal depression, Interpersonal counselling, Randomised controlled trial, Feasibility study

## Abstract

**Background:**

Up to one in eight women experience depression during pregnancy. In the UK, low intensity cognitive behavioural therapy (CBT) is the main psychological treatment offered for those with mild or moderate depression and is recommended during the perinatal period, however referral by midwives and take up of treatment by pregnant women is extremely low.

Interpersonal Counselling (IPC) is a brief, low-intensity form of Interpersonal Psychotherapy (IPT) that focuses on areas of concern to service users during pregnancy.

To improve psychological treatment for depression during pregnancy, the study aimed to assess the feasibility and acceptability of a trial of IPC for antenatal depression in routine NHS services compared to low intensity perinatal specific CBT.

**Methods:**

We conducted a small randomised controlled trial in two centres. A total of 52 pregnant women with mild or moderate depression were randomised to receive 6 sessions of IPC or perinatal specific CBT. Treatment was provided by 12 junior mental health workers (jMHW). The primary outcome was the number of women recruited to the point of randomisation. Secondary outcomes included maternal mood, couple functioning, attachment, functioning, treatment adherence, and participant and staff acceptability.

**Results:**

The study was feasible and acceptable. Recruitment was successful through scanning clinics, only 6 of the 52 women were recruited through midwives. 71% of women in IPC completed treatment. Women reported IPC was acceptable, and supervisors reported high treatment competence in IPC arm by jMHWs. Outcome measures indicated there was improvement in mood in both groups (Change in EPDS score IPC 4.4 (s.d. 5.1) and CBT 4.0 (s.d. 4.8).

**Conclusions:**

This was a feasibility study and was not large enough to detect important differences between IPC and perinatal specific CBT. A full-scale trial of IPC for antenatal depression in routine IAPT services is feasible.

**Trial registration:**

This study has been registered with ISRCTN registry 11513120. – date of registration 05/04/2018.

**Supplementary Information:**

The online version contains supplementary material available at 10.1186/s12888-021-03482-x.

## Background

Antenatal depression is common, with a point prevalence of up to 12.9% [1]. There are known to be adverse effects on the mother and foetus with increasing evidence to show that antenatal depression is associated with a range of poor outcomes from premature birth [2], to behavioural problems in infancy, and depression in adolescence (Stein et al. [3]). There is very limited evidence for the effectiveness of psychological interventions for antenatal depression [4]. This is despite the fact that there is a widespread reluctance of mothers to take antidepressants during pregnancy and concern amongst clinicians about prescribing them, meaning that psychological interventions are particularly important at this time [5].

The current treatment recommendation for mild to moderate depression during pregnancy, is supported self-help approach using the principles of cognitive behavioural therapy (CBT) [6]. However, the provision and take up of CBT treatment for antenatal depression is low. Women who are pregnant receive lower rates of mental health interventions than those outside the perinatal period (30% vs 50%), and most of these treatments involve medication (McManus et al. [7]). This is concerning given the worries pregnant women have about medication. Furthermore, there are important limitations of CBT for pregnancy as a model for less experienced therapists because: 1) without adaptation and additional training, CBT has few explicit strategies to manage many of the problems that are common for women with antenatal depression, including role transitions and problems in communication. This is critical as conflict in relationships and poor social support are the strongest risk factors for antenatal depression (Lancaster [8]). 2) low intensity CBT does not manage problems with grief (i.e., miscarriage, still-birth, termination, loss of parents at time of birth of child) that contributes to depressive symptoms. 3) CBT is not designed to involve the partner.

In contrast, Interpersonal Counselling (IPC) a brief treatment derived from Interpersonal Therapy (IPT) is based on relational theory. It acknowledges that although depression is multicausal, a key precipitating factor occurs when problems in interpersonal relationships trigger symptoms of depression, such as low mood or sleeplessness, and these symptoms further compromise these relationships. It uniquely focuses on approaches that help manage changes in role and losses (e.g., miscarriage, still-birth, termination, previous loss of would be grandparents) and the impact of these on relationships and mood. By directly approaching these issues, IPC addresses what service users report are significant worries and concerns for them during pregnancy. Furthermore, the brief nature of IPC makes it appropriate for mild to moderate forms of depression, and can be delivered by those without specialist mental health training, thus making acceptable and appropriate psychological interventions for pregnant women more widely available.

IPC has been clearly developed and manualised as an intervention allowing assessment of fidelity to the model. Although there have been very few studies of the effectiveness of IPC and none in pregnancy, one study of depression in primary care in Italy found it to be more effective than antidepressants (selective serotonin reuptake inhibitors) particularly for those with less severe depression [9]. A small feasibility study of IPC for antenatal depression in the US amongst low income mothers indicated high satisfaction with IPC and some improvement in mood [10]. A more intensive intervention, a brief form of IPT, offered in pregnancy and into the first year postnatally showed some promise in improving outcomes in difficult to engage low-income women in the US [11].

There are therefore good reasons to hypothesise that this form of therapy would be more acceptable during the antenatal period and particularly effective in treating depression at this time. We aimed to test the hypothesis that a large scale RCT of IPC in routine NHS services is feasible.

## Methods

### Study design and objectives

This phase II trial aimed to established the feasibility of a larger scale Phase III pragmatic Randomised Controlled Trial (RCT) of IPC versus CBT for mild to moderate antenatal depression offered within routine National Health Service psychological treatment services conducted across two centres in the United Kingdom (Bristol and Exeter). Full trial protocol is available at https://research-information.bris.ac.uk/ws/portalfiles/portal/207837164/Full_text_PDF_final_published_version_.pdf

### Training

The intervention was delivered by junior mental health workers (in England called Psychological Wellbeing Practitioners) working in Improving Access to Psychological Therapies (IAPT) services. They are graduates from any discipline who have undertaken 45 days of academic training in an accredited psychological treatments course, alongside supervised practice in low intensity CBT, over one academic year in order to become ‘Psychological Wellbeing Practitioners’.

They were randomised to receive either 3 days training in IPC or 1 day CBT top-up with a focus on perinatal-specific guided self-help. Competence in the IPC model was rated by supervisors for each of the six junior mental health workers delivering IPC.

### Intervention

IPC involved up to six 30–45 min sessions, with the option of inviting a partner or significant other to one of the sessions. The tasks of IPC included identifying symptoms of depression and relating these to interpersonal problems, identifying protective factors and vulnerabilities, agreeing the focus and identifying who will assist in promoting recovery. IPC is structured around one of four focal areas; grief, role transitions, role disputes or interpersonal sensitivities. The focal area is collaboratively chosen to address the primary interpersonal context in which depression occurs and common strategies focusing on improving communication and processing emotions are combined with focus specific interventions e.g. mourning the loss of a significant role or identifying key issues and different expectations in a dispute. The individual’s social network is actively engaged and guided to understand and contribute to recovery from depression. Supervision of jMHW was provided by experienced IPT practitioners, by telephone on a fortnightly basis. PWPs were required to present their casework and were provided with written and verbal feedback on competence in the model. Supervision on 1–2 training cases was conducted in a group format, followed by individual supervision on trial casework.

### Comparison

Brief CBT was provided by the junior mental health workers. This was the usual care offered of up to six sessions of individual sessions involving guided self-help based on CBT principles. In addition, for the purposes of this trial the junior mental health workers offering this approach, attended a 1 day training on mental health during perinatal period. As such, we have called this perinatally enhanced brief-CBT. Supervision was carried out fortnightly in a group setting as part of the routine service provided.

### Participants

Participants were women, 18 years or over, between 10 and 24 weeks of pregnancy, with an Edinburgh Depression Scale (EPDS) [12] score 10 or above and had ICD-10 mild or moderate depression determined by the Clinical Interview Schedule Revised (CIS-R) [13], a structured computerised psychiatric interview that provides diagnostic information.

Women were excluded if they reported having: a psychotic illness, an organic brain disorder, bipolar disorder, personality disorder, alcohol or substance dependency. Also excluded were those at high suicide risk in the assessor’s judgement or from their response to items on suicide on the CIS-R or EPDS, those meeting criteria for severe depression (ICD-10) according to CIS-R, and those who had received CBT or IPT within the last 6 months.

### Recruitment

This took place between 1st January 2019 and 30th September 2019. We aimed to compare the feasibility of two different recruitment methods:

Method 1: Midwife booking clinics. Women who screened positive on a two-item depression case finding instrument [14] used routinely by midwives at their first appointment with the midwife (around 12 weeks of pregnancy) and who consented to be contacted were referred to the research team.

Method 2: Women at the ultrasound scan clinics (12 and 20 week scans) were also given study information, screening questions (EPDS score with 10 or above a positive screen) and a consent to contact form from either a research assistant or administrative assistant staff.

Eligible women were then asked to complete a face-to-face assessment with a research assistant to establish eligibility and obtain consent for the study and collect baseline data. Partners were recruited in person at this baseline visit, online or by mail to complete measurements of their mood. Randomisation will be carried out remotely by Bristol Randomised Trials Collaboration randomisation service. Randomisation was 1:1 and stratified by recruiting centre and minimised by parity (with random block sizes). Follow-up data at 12 weeks post randomisation were collected online.

Following randomisation women received a routine clinical assessment with the IAPT service and those who were considered to be clinically appropriate for a low intensity intervention with the IAPT service, were allocated to a PWP for either IPC or perinatal-specific CBT. Both treatments were low intensity individual psychological interventions of up to six sessions each between 30 and 45 min long. This routine assessment was a service requirement as part of the contract with the psychological treatment service.

### Assessments

The assessment measures at baseline measured mood, relationship quality and quality of life. The CIS-R a computerised structured diagnostic interview [13] was completed at baseline only. This provided and ICD-10 diagnosis and confirmed eligibility to the study (mild or moderate depression). The EPDS [12] measures mood symptoms and has been validated and widely used in the perinatal period. The revised Dyadic adjustment scale [15] which assesses partner satisfaction and the maternal antenatal attachment scale which measures the relationship between the mother and her unborn baby [16], have both been validated during pregnancy. The EQ-5D-5L is a generic measure of health [17] widely used in clinical trials to allow health economic evaluations. The ReQuol-10 was developed as a mental health specific quality of life measure which has shown good validity against other measures of quality of life [18].

These measures were completed at baseline and 12 weeks post randomisation with the exception of the CIS-R which was completed at baseline only.

All 12-week measures were collected on-line with participants prompted via e mail messages. In addition, at 12-weeks, data were collected from the jMHWs on the number of sessions attended, number of sessions with a partner, whether therapy was considered by the jMHW complete, if individuals were provided with a referral for additional treatment at a higher intensity within the service. At the end of the study, supervisors rated each jMHW using a four-item checklist assessing their ability to follow the IPC model.

### Data analyses

As this is a feasibility study, it was not powered for statistical testing of any difference between the interventions. The analyses focus on reporting data that will be used for planning and assessing the feasibility of the full scale trial. Feasibility measures reported include the number and proportions of eligible, recruited, randomised, started treatment, completed treatment, lost to follow-up, completing follow-up and completing individual outcome measures. The primary outcome was the number of eligible women successfully recruited to the point of randomisation. We set a recruitment target of 60 subjects from 338 potentially eligible women (17.8%) during the nine-month recruitment period giving a 95% confidence interval for recruitment of (13.9, 22.3%).

Descriptive statistics for clinical and health economic outcome measures considered for the main trial are reported according to allocation to IPC or CBT .

### Nested qualitative study

We assessed the acceptability of the recruitment method, intervention and study design through a series of in-depth interviews with participants and staff. Women who received therapy (both IPC and CBT) were interviewed and those who dropped out of the process before therapy. A few partners; the jMHWs who were trained in IPC; and the managers from both services were also interviewed. Interview topic guides were informed by the research literature, team discussions and input from our PPI groups. Interviews were recorded, transcribed verbatim by a professional transcription service and anonymised. Thematic analysis of the data was an ongoing and iterative process using NVivo software to organise and code the transcripts. (QSR International Pty Ltd [19]) Codes and themes were developed and discussed within the qualitative research team at regular intervals during both data collection and analysis, to achieve consensus ([20]).

## Results

### Recruitment of services and training

The local IAPT services in Bristol and Exeter agreed to participate and 12 jMHWs were allocated randomly, six to IPC training and six to perinatal-specific CBT training.

### Recruitment of women during pregnancy and outcome data collection

**Fig. 1:**
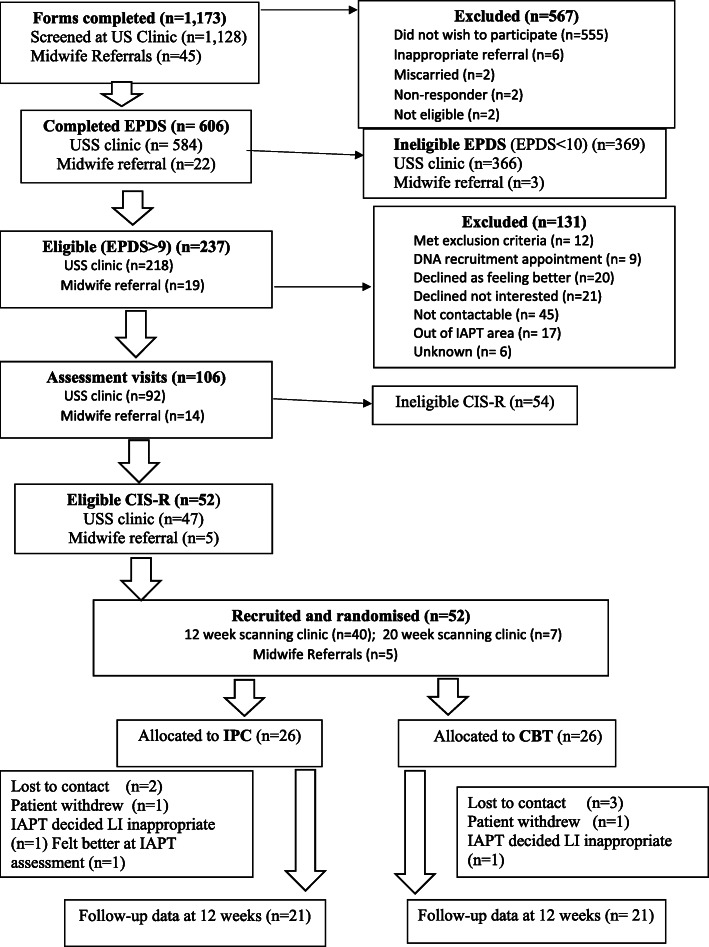
CONSORT Flow Diagram

A total of 1173 potentially eligible women were approached during the 9-month recruitment period with 1128 through the scanning clinics and 45 through midwives. (see Consort diagram Fig. 1) Of the 606 who completed the EPDS, 237 (39%) had an EPDS score of 10 or more. Of these 237 potentially eligible women, 106 (44%) were assessed at baseline and of these 52 (49%) were eligible, meeting all criteria for the study including a diagnosis of mild or moderate depression according to the CIS-R; they were randomised to IPC or perinatal-specific CBT. Of the 52 randomised, 42 (81%) provided outcome data at 12 weeks post randomisation. Only 3 of the 52 randomised were not considered appropriate for low intensity psychological intervention following routine clinical assessment by the service (one because they had improved by the time the assessment was carried out).

The baseline characteristics are shown in Table 1. Women were recruited at a median of 15 weeks of pregnancy and had a mean age of 31.4 and 54% were primiparous. It is notable that there was a selection bias according to educational level with 73%, having a degree.

**Table 1:** Baseline and Demographic Characteristics of the Sample

	CBT ***N*** = 26	IPC ***N*** = 26	Overall ***N*** = 52
**Age, Mean (Std)**	30.1 (4.7)	32.8 (4.6)	31.4 (4.8)
**No. of weeks pregnant, Median (Min, Max)**	14 (12, 26)	16.5 (13, 23)	15 (12, 26)
**First Pregnancy, N (%)**	14 (54%)	14 (54%)	28 (54%)
**Ethnic Group, N (%)**			
White	22 (85%)	25 (96%)	47 (90%)
Mixed/ multiple ethnic groups	2 (8%)	0	2 (4%)
Asian/ Asian British	0	0	0
Black/ African/ Caribbean/ Black British	1 (4%)	0	1 (2%)
Other Ethnic Group	1 (4%)	1 (4%)	2 (4%)
**Highest Educational Qualification N (%)**			
No formal qualification	2 (8%)	0	2 (4%)
GCSE/ CSE/ O Level	2 (8%)	0	2 (4%)
A-Level/ AS level	5 (19%)	3 (12%)	8 (15%)
Degree	17 (65%)	21 (81%)	38 (73%)
Other	0	2 (8%)	2 (4%)
**Marital Status, N (%)**			
Married/ registered civil partnership	14 (54%)	12 (46%)	26 (50%)
Living together	8 (31%)	14 (54%)	22 (42%)
Single	4 (15%)	0	4 (8%)
Widowed/ Divorced/ Separated	0	0	0
**No. aged 18+ living in household, N (%)**			
0	1 (4%)	0	1 (2%)
1	23 (88%)	24 (92%)	47 (90%)
2	2 (8%)	2 (8%)	4 (8%)
**Employment, N (%)**			
In paid work (full or part time)	21 (81%)	26 (100%)	47 (90%)
Unemployed	2 (8%)	0	2 (4%)
Looking after family or home	0	0	0
Unable to work	1 (4%)	0	1 (2%)
Full-time education/ training	2 (8%)	0	2 (4%)
EPDS > 12, N (%)	20 (77%)	23 (88%)	43 (83%)
CISR Diagnosis			
Mild Depression (F32.0) N (%)	9 (35%)	7 (27%)	16 (31%)
Moderate Depression (F32.1) N (%)	15 (58%)	17 (65%)	32 (62%)
Mixed anxiety and depression (F41.2) or	0	1 (4%)	1 (2%)
Specific Phobias (F40.2) N (%) (***recruited in error***)^a^	2 (4%	1 (4%)	3 (6%)
RDAS Scale, Median (IQR)	*N* = 25 50 (42, 54)	*N* = 26 51 (42, 56)	*N* = 51 51 (42, 55)
MAAS Scale, Mean (Std)	68.5 (11.5)	71.4 (9.0)	69.9 (10.3)
EQ-5D-5L, Mean (Std)	0.730 (0.08)	0.701 (0.13)	0.716 (0.11)
ReQol, Mean (Std)	24.5 (4.6)	24.8 (4.6)	24.7 (4.5)

(foot note: *EPDS* Edinburgh Postnatal Depression Scale, *CIS-R* clinical interview schedule revised *RDAS* Revised dyadic adjustment scale, *MAAS* Maternal Antenatal attachment scale, *EQ-5D-5L* Euroquol measure of quality of life, version 5 L, *ReQuol-10* Recovering quality of life questionnaire.) ^a^Not mild or moderate depression on CIS-R so should not have been included, recruited in error by research team

As very few partners were recruited to the study (*n* = 13), data are omitted for simplicity.

### Outcomes

The outcomes relevant to the feasibility of the trial are shown in Table 2. In summary, most recruits came though scanning clinics compared to midwife booking clinics. Of the 26 randomised to IPC, 2 started CBT as timing was more convenient to them. Of the 17 who started IPC, 12 (71%) completed five or more sessions and were considered to have completed treatment. Of the 52 women randomised, two women, one from each study arm, were stepped up to a higher intensity intervention after completing treatment.

**Table 2:** Recruitment and Retention

Recruitment	N, %	95% Confidence Interval
Potentially Eligible/ Forms returned or referred		
By midwife	19/45 (42%)	(28, 58%)
At Scanning clinics	218/1128 (19%)	(17, 22%)
**Total 237/1173**	**(20%)**	**(18, 23%)**
Recruited / Potentially Eligible		
By midwife	6/19 (32%)	(13, 57%)
At scanning clinics	46/218 (21%) (38 at 12 week scan, 8 at 20 week scan)	(16, 27%)
**Total**	52/237 (22%)	(17, 28%)
Recruited/ Randomised (Total)	52/52 (100%)	(93, 100%)
**Treatment**		
Randomised/ Clinically Eligible following IAPT assessment	49/52 (94%)	(84, 99%)
Randomised to CBT/ Started CBT	15/26 (58%)	**(37, 77%)**
Randomised to IPC / Started IPC	17/26 (65%)	**(44, 83%)**
Randomised to IPC/ Started CBT	2/26 (8%)	**(1, 25%)**
Started IPC / Completed course of treatment to an adequate level	12/17 (71%)	(44, 90%)
Randomised to CBT/ Required step-up to high intensity treatment	1/26 (4%)	(0.1, 20%)
Randomised to IPC/ Required step-up to high intensity treatment	1/26 (4%)	(0.1, 20%)
**Follow up data**		
Randomised/ Completed 12 week EPDS Follow-up	42/52 (81%)	(67, 90%)
Randomised/ Provided all 12 week Follow-up measures with no missing data	37 (71%) (95% CI: 57, 83%) completed all follow-up measures.	

The clinical and health economic outcomes at 12 weeks are shown in Table 3. In summary, 81% women provided primary outcome data at 12 weeks post randomisation. For the group randomised to IPC, the mean EPDS score at 12 weeks post randomisation was 10.7 (s.d. 3.9) and for those randomised to CBT it was 11.5 (s.d. 4.3). Both groups recorded an improvement in depressive symptoms compared to baseline, the mean drop in EPDS score was 4.4 (s.d. 5.1) for the IPC group and 4.0 (s.d. 4.8) for the CBT group.

**Table 3:** Follow-up Data collected from the sample

	IPC	CBT	Overall
No. of weeks from recruitment to starting treatment, Median (IQR)	*N* = 19 5.0 (3.6, 7.1)	*N* = 15 7.1 (4.0, 9.0)	*N* = 34 5.0 (4.0, 8.3)
No. of weeks from recruitment when follow-up completed, Median (IQR)	*N* = 21 13.1 (12.7, 14.1)	*N* = 21 14.4 (13.0, 16.0)	*N* = 42 13.9 (12.7, 15.3)
Total Number of sessions attended, Median (IQR)	*N* = 19 6 (4, 6)	*N* = 15 6 (3, 7)	*N* = 34 6 (4, 6)
No. of partners attending 1 or more sessions, N (%)	3 (16%)	0 (0%)^a^	
EPDS, Mean (Std	10.7 (3.9) *N* = 21	11.5 (4.3) *N* = 21	11.1 (4.1) *N* = 42
Change in EPDS score from baseline to 12 week follow-up, Mean (Std)	4.4 (5.1)	4.0 (4.8)	4.2 (4.9)
EPDS 9/10 cut-off, N(%) with a score of 10+	12 (57%)	14 (67%)	26 (62%)
EPDS 12/13 cut-off, N(%) with a score of 13+	6 (29%)	8 (38%)	14 (33%)
RDAS Scale, Median (IQR)	51 (48, 56) *N* = 21	52 (48, 55) *N* = 19	52 (48, 55.5) *N* = 40
MAAS Scale, Mean (Std)	77.9 (5.6) *N* = 20	76.6 (7.7) *N* = 20	77.2 (6.7) *N* = 40
EQ-5D-5L, Mean (Std)	0.666 (0.169)	0.701 (0.11)	0.683 (0.195)
ReQoL-10, Mean (Std)	27.2 (6.0) *N* = 20	27.9 (6.8) *N* = 19	27.5 (6.3) *N* = 39
ReQoL-10 Change Score, Mean (Std)	1.9 (5.5)	2.9 (5.4)	2.4 (5.4)
Medication use to improve mental health (Yes/No)	0	0	0
Resource use (societal), mean GBP£ (Std)	462.83 (1106)	412.38 (719)	438.29 (926)

^a^partners are not invited in routine low intensity CBT model

### Health economic measures

Response rates for all economic measures were high enough to be confident of collecting the necessary data needed to undertake an economic evaluation alongside a future definitive trial.

Both groups recorded an improvement in health-related quality of life (HRQoL) as measured by ReQoL-10 a measure developed to assess the quality of life of people with different mental health conditions. The mean increase in ReQoL-10 score was 1.9 (s.d. 5.5) for the IPC group and 2.9 (s.d. 5.4) for the CBT group.

Both groups recorded a drop in HRQoL scores as measured by EQ-5D-5L the NICE recommended QoL measure for economic evaluation which captures both mental and physical attributes of QoL. The mean change in EQ-5D-5L utility scores was − 0.038 (s.d. 0.16) for the IPC group and − 0.031 (s.d. 0.087) for the CBT group. Scores fell due to the natural decline in the physical health domains as gestation increases, however this fall was lessened by increased scores in the mental health domain.

The Resource Use Questionnaire (RUQ) was costed providing a total cost for both treatment and non-treatment related costs. The total cost per patient including treatment for IPC was £774 (s.d. £1032) and for CBT £539 (s.d. £492).

#### Acceptability from qualitative interviews

Twenty-three women were interviewed for the nested qualitative study: 19 who received therapy and four who dropped out before therapy. Three partners were interviewed; six jMHWs who were trained in IPC; and the managers from both services.

Overall, all participants and partners interviewed found the study screening and recruitment process highly acceptable including the completion of the outcome measures online.

Several women found it quite difficult to understand their emotions in early pregnancy with many finding it an anxious time. Those who had no previous experience of mental health problems, reported being reluctant to discuss such issues with a midwife at their booking appointment, so “*this study was a lifeline”* for them.

Scanning clinics were felt to be a good place to read the study leaflet, with time in the waiting room to think about it or discuss with a partner. It gave them “*space to acknowledge their low mood”.* It was also a more confidential way of admitting to low mood than talking to a midwife.

Most of those interviewed understood the concept of randomisation; a few expressed an expectation that the allocated therapy would be the *“right one for them”*. The recruitment appointment also enabled them to “*acknowledge that things were not right”* for them.

Psychological services “fast-tracked” pregnant women (ie. saw them within 4 weeks of referral), and many were pleased with how quickly and efficiently their sessions were organised. However, women were reluctant to take time off work to attend sessions and combined with limited provision of ‘out of hours’ sessions and restricted venue options, they sometimes had to wait for several weeks before starting their treatment course.

Most women liked seeing their therapist face-to-face, and for some the commitment of having an appointment to attend outside the home, motivated attendance. Those who received telephone-only therapy reported that a lack of rapport meant that it took longer to ‘open up’ or ‘get to know’ their therapist and feel relaxed. Most agreed that offering a combination of face-to-face and telephone sessions would be the most acceptable method of service provision. There were no video sessions at the time of the study.

Women found both treatments focused on practical issues, offered ‘tools for life’ and they appreciated being given exercises to complete and handouts that they could refer to later.

Women described IPC as being “*helpful*”,“*practical*” and empowered them to *“ask for help”*. They particularly mentioned goal setting, mapping their circle of support and communication skills with partner as being very useful. CBT had a more structured and task-based therapist-led approach which was appreciated by some women and they were generally happy with it.

*Partners*: There were many reasons why partners did not attend IPC sessions as intended. jMHWs were not used to conducting joint sessions and felt uncomfortable with the idea, but also women did not always feel it was relevant to ask them, whether due to work issues, needing them to provide childcare or just not being happy with the idea themselves. However, those men who were interviewed saw the intervention as positive and helpful particularly for relationships.

At first, some jMHWs felt under-prepared and anxious when delivering IPC therapy, which was a new treatment to them. However, they felt that IPC was a very appropriate therapy for pregnant women especially concerning partners and relationships, and found the IPC approach ‘more human’. JMHWs reported that it was sometimes difficult to keep sessions to their allotted length, but felt they learnt much from it and gained in confidence throughout the study (see Table 4).

**Table 4:** Acceptability of trial; quotes from qualitative interviews

**‘*Study was a lifeline’*.**	
*I think this study is awesome because the therapy I had was amazing and it made a huge difference to my mood and it made me feel so much better about my pregnancy and so much happier, and I think if that hadn’t been there I don’t know how that would have been addressed again…. (1006, site A, primip)*	
**Scanning clinics:** ***‘Space to acknowledge low mood’***	
*That was a good time for me actually to have it, yeah, and actually it’s quite good if you’re giving something while you’re in a waiting area because it’s something to do while you’re waiting rather than when you’re rushing around, so it was quite a good time to look at it. (1034, site A, primip)*	
*…it might not have been something I spoke to my midwife about, I might not have….if I hadn’t been directly asked I might not have answered those questions in that respect. This was a confidential way […] that I could do it in my own time to refSlect in how I was feeling’ (1021, site A, multip)*	
**The recruitment appointment:** ***‘Recognising things are not right’.***	
*It felt good to just talk about how I had been feeling with someone, actually. I know that you were doing it for the purpose of finding out which help I needed, but even sometimes just sharing your thoughts with someone can help. I have to admit I was nervous about any implications, I was worried whether I would come across as a bad mum or whether it would lead to any intervention from any government bodies, but overall it felt good to be able to share how I was feeling with someone. (1025, site A, primip)*	
**Timing of therapy:** ***‘treatment to start as early as possible’.***	
*I remember having my first session with the therapist and then she had some time off or holiday, so there was a bit of a gap between my first and second session,… It was quite difficult juggling it and fitting it in if I’m honest, and in fact I haven’t got on very well with getting to the sessions because I have either been really late or I’ve had problems with childcare. So quite a slow process. (1029, site A, multip, IPC)*	
**Delivery mode:** ***‘face to face is good’***	
*I think face to face is obviously better, you’ve got your eye contact and it just feels different I think face to face, but I am sure telephone will be more helpful than not having it. But I would say face to face is preferable, just to have that rapport as well with the therapist I think helps.(1034, site A, primip, IPC)*	
**Content of IPC therapy:** ***asking for help***	
*Definitely, I am much better at accepting help now, and to the point where I am trying to make my husband also better at accepting help, because we needed help, and it’s baby steps that I do feel like I’ve come a long way. (1018, site A, multip, IPC)*	
*…knowing who is in the support group and [name] reflected on that actually, and there was a chart that she gave me where I could write who it was, my relationship with them, and what good they bring me, and can I rely on them for emotional and physical support, that was really helpful to go through, to know who I had and who I could rely on. (2005, site B, primip, IPC)*	
**Junior Mental Health Worker views** ***“IPC approach is ‘more human”***	
*There was freedom and a different focus [in IPC therapy], focus still on depression but there was focus on relationship that it’s not really the main thing in CBT, and I think a lot of clients that I worked with found that helpful and having space to talk about things a bit more freely it seemed like it was helpful. (jMHW, Site A)*	
*Different [from CBT]… I think the relationship aspect of it was really helpful, who’s in the networks and who is going to support you and… what are you going to do to help… what skills can we develop to help you get that… express your needs, I find that so helpful, especially for pregnant women, and also new babies as well (jMHW site B)*	

## Discussion

This is the first study to assess the feasibility of a trial and acceptability of IPC for antenatal depression. We found that the trial was both feasible and acceptable.

We found that recruitment using the routine pathway through midwives is not feasible but recruitment through scanning clinics is both feasible and acceptable to women. This raises important questions about the identification of less severe mental health problems in maternity services. Currently, midwives ask the Whooley questions face-to-face to identify women who may have antenatal depression [6]. This screening method has high sensitivity (95%) and modest specificity (65%) when used as a self-report tool for antenatal depression [21]. However, using these questions face-to-face has a much lower sensitivity with lower depression detection (7.5%) when used in midwife run booking clinics and thus low referral rates in this study. This low rate of detection was also reported in another recent UK study (10%, Howard et al. 2018). Anecdotal evidence suggests that women are not always aware that they can be referred for further help following a positive Whooley screen [22]. In contrast women were willing to complete a depression scale whilst waiting in the scanning clinic and around 20% of those who completed the scale scored above the threshold indicating at least mild depression. This rate is consistent with other studies using the EPDS [23]. Therefore, we will recruit from scanning clinics in a full-scale trial of IPC.

IPC is a recently developed brief intervention derived from a more intensive treatment, Interpersonal therapy (IPT). It focuses on the importance of relationships in the onset and maintenance of depression and addresses issues that are reported by women to be particularly important during pregnancy. Although a trial of the more intensive IPT has shown good effect for postnatal depression when delivered by telephone by specially trained nurses (Dennis et al. 2020), we have shown here that IPC, can be delivered in routine psychological treatment services. We have also shown that the intervention can be taught to junior mental health practitioners without extensive previous clinical training following 3 days training and with supervision of cases. Although there was some anxiety about learning a new intervention, they found it very relevant to the perinatal context and quickly gained in confidence in delivering IPC throughout the 9-month course of treatment provision. This was echoed by the women who received the intervention who found particular aspects of the intervention helpful. Although very few partners completed questionnaires or attended sessions, those partners who were interviewed, saw the intervention as positive and helpful particularly for relationships.

### Limitations

There was considerable drop out between randomisation and delivering the intervention with only 65% of those randomised starting a course of treatment. The main reason for this was that the psychological treatment service was unable to contact those who were referred. There is a practical question about how persistent this attempt to contact women should be. There is concern in psychological treatment services that those who do not respond to correspondence or telephone contact are unmotivated and therefore unlikely to attend or benefit from a psychological intervention. However, depression is demotivating and there is evidence that an assertive approach may be needed in some groups to engage them in treatment [24]. A more assertive approach may be needed along with the prioritisation of perinatal women to the service to improve treatment up take.

Although we aimed to complete the intervention and the outcome measures before the postnatal period, for practical reasons in larger study this may not always be possible for those recruited up to 26 weeks of pregnancy. Follow up assessments may be more difficult to collect during the postnatal period and transient mood changes in the first postnatal week could effect outcome measures.

We were not powered to detect a difference in outcome, and there was no evidence of difference between the two interventions. It is worth noting that the therapists in the comparison group received specific perinatal training at the time of the intervention and a more pragmatic real-world design would compare IPC to standard usual care in psychological treatment services.

## Conclusion

A trial of IPC taught to junior mental health workers in routine psychological treatment services in the UK is feasible and acceptable to women and staff. The progression criteria were met (see on-line supplement) and a full scale trial is now needed to evaluate the effectiveness and cost effectiveness of IPC for mild or moderate antenatal depression.

## Supplementary Information

**Additional file 1.**

## Data Availability

The datasets used and/or analysed during the current study are available from the corresponding author on reasonable request.

## Abbreviations

CBT: Cognitive behavioural therapy
IPC: Interpersonal counselling
IPT: Interpersonal psychotherapy
jMHW: Junior mental health worker
EPDS: Edinburgh postnatal depression scale
IAPT: Improving access to psychological treatment
CIS-R: Clinical interview schedule revised
EQ-5D-5L: Euroquol measure of quality of life, version 5 L
ReQuol-10: Recovering quality of life questionnaire
RDAS: Revised dyadic adjustment scale
MAAS: Maternal antenatal attachment scale
HRQoL: Health-related quality of life
