# Supplementary material for: Interpersonal counselling versus perinatal-specific cognitive behavioural therapy for women with depression during pregnancy offered in routine psychological treatment services: a phase II randomised trial
Source: BMC Psychiatry. 2021 Oct 15;21:504. doi: 10.1186/s12888-021-03482-x (PMC8518253; doi:10.1186/s12888-021-03482-x)
Supplement: Supplementary file 1 — Additional file 1. [file 12888_2021_3482_MOESM1_ESM.docx]

Progression criteria met at the end of the trial:

**Interpersonal counselling versus perinatal-specific cognitive behavioural therapy for women with depression during pregnancy offered in routine psychological treatment services: A phase II trial.**

These progression criteria were agreed with both the funders, the National Institute for Health Research UK, and the trial steering committee:

1) An average of 6 participants per month (across both sites) are recruited and randomised during the last 4 months of recruitment.

2) At least 75% of women randomised provide complete outcome data.

3) At least two-thirds of women randomised to, and who start IPC therapy, receive complete therapy as judged by the PWP.

4) IPC and trial design is considered broadly acceptable from in-depth interviews.

5) Practitioners are judged by their supervisors to be following the IPC model adequately using an agreed checklist.
